# Membranous-like glomerulopathy with masked IgG-k deposits in a pediatric patient with juvenile idiopathic arthritis

**DOI:** 10.1007/s00467-025-06954-4

**Published:** 2025-09-15

**Authors:** Priyanka Chati, Jill Krissberg, Kammi Henriksen, Brian Nolan, Meredith Harris

**Affiliations:** 1 Division of Nephrology, Ann & Robert H. Lurie Children’s Hospital, Chicago, IL USA; 2 Division of Rheumatology, Ann & Robert H. Lurie Children’s Hospital, Chicago, IL USA; 3 Division of Pathology, University of Chicago, Chicago, IL USA; 4 Division of Nephrology, Cincinnati Children’s Hospital Medical Center, Cincinnati, OH USA

**Keywords:** Membranous nephropathy, Masked monoclonal deposits, Juvenile idiopathic arthritis, Proteinuria, C3-glomerulonephritis

## Abstract

Membranous-like glomerulopathy with masked IgG-kappa deposits (MGMID) is a rare entity described primarily among young females with previously diagnosed autoimmune diseases. We present a 12-year-old female with systemic juvenile idiopathic arthritis (sJIA) with persistent non-nephrotic range proteinuria despite normal kidney function. She underwent two kidney biopsies with the second ultimately confirming her diagnosis. The initial biopsy was suggestive of mild C3 glomerulonephritis (C3GN). She was started on an angiotensin-converting enzyme inhibitor (ACE-I) without improvement. Proteinuria progressed to the nephrotic range, prompting initiation of high-dose steroids followed by a steroid taper. Mycophenolate was added during steroid weaning due to ongoing proteinuria. Despite full-dose mycophenolate and ACE-I therapy, a repeat biopsy was performed due to lack of response and revealed MGMID. She remains on full-dose mycophenolate and lisinopril with significant improvement in her proteinuria.

## Background

Membranous-like glomerulopathy with masked IgG-kappa deposits is a rare, biopsy-diagnosed condition predominantly affecting young females under the age of 40, with a mean reported age of 26 [1–4]. Patients typically present with proteinuria, with or without microscopic hematuria, and often have negative serologic workup [3]. A family or personal history is typically significant for autoimmune disease, particularly systemic lupus erythematosus or Sjögren’s syndrome; however, a vague autoimmune phenomenon has also been described [1, 3, 4]. Histological diagnosis relies on findings from light microscopy (LM), immunofluorescence (IF), and electron microscopy (EM). In MGMID, EM typically shows subepithelial deposits, while routine IF reveals isolated C3 staining. This pattern often leads to misdiagnosis of C3 glomerulonephritis (C3GN) or infection-related glomerulonephritis [3, 4]. Confirming the diagnosis requires repeat IF using formalin-fixed, paraffin-embedded tissue with protease digestion, which “unmasks” the IgG-kappa deposits [1]. Identifying the correct diagnosis is important for therapeutic decision-making, as the treatment for C3GN can be more aggressive to control complement overactivation when compared to treatment for MGMID.

## Case report

A 12-year-old Hispanic female with well-controlled systemic juvenile idiopathic arthritis (sJIA) complicated by hypertension, posterior reversible encephalopathy syndrome, and acute anterior uveitis, on biweekly tocilizumab, was referred to the nephrology clinic for persistent proteinuria. She was diagnosed with sJIA in 2022 and was in clinical remission on tocilizumab several months later. Proteinuria was first noted in 2023, 1.5 years after diagnosis of sJIA and 11 months into a state of clinically inactive disease.

She was referred to nephrology for proteinuria in 2024 with urine protein-to-creatinine ratio (UPC) ranging from 1 to 1.5 mg/mg (normal < 0.2 g/g) and a normal albumin of 4 g/dL. She had no microscopic hematuria, and her serum creatinine was normal at 0.49 mg/dL with an estimated glomerular filtration rate (eGFR) of 104.5 mL/min/1.73 m^2^ using the CKiD U25 equation. She was normotensive with a blood pressure of 99/70. Serologic workup was largely unremarkable aside from a positive antinuclear antibody titer (ANA) at 1:640 and a mildly low C3 level at 73 mg/dL (reference range 93–203 mg/dL). At the time of presentation with proteinuria, the patient’s sJIA was clinically inactive, with no fevers, rash, or arthritis, and normal inflammatory markers (ESR/CRP).

Initial kidney biopsy revealed mild C3GN versus latent infection-related GN. The sample contained 15–22 glomeruli, of which 1–3 were globally sclerosed. The remaining glomeruli showed mild segmental mesangial hypercellularity. Focal mild interstitial fibrosis and tubular atrophy (IFTA) were seen without significant interstitial inflammation. IF microscopy showed 5 glomeruli, 1 of which was globally sclerosed. Tubulointerstitial changes were similar to LM findings noted above. IF showed mesangial staining for C3 (1–2 +). No glomerular staining for IgG, IgA, IgM, C1q, or kappa or lambda light chains was seen. EM revealed parts of 3 glomeruli, 2 of which were globally sclerosed. A few mesangial electron-dense deposits and a single subepithelial electron-dense deposit were seen and identified as normal. The podocyte foot processes showed focal effacement. Serum C3 normalized on repeat testing one month later to 99.6 mg/dL. The C3 Glomerulopathy Complement Panel (C3G-CP) was sent to Iowa Health Care and was negative.

She was started on angiotensin-converting enzyme inhibitor (ACE-I) therapy for two months, yet UPC increased into the nephrotic range (2–2.2 mg/mg). High-dose prednisone (60 mg daily) was initiated for 4 weeks, resulting in mild improvement of her UPC to 1.4 mg/mg. Steroids were tapered, and mycophenolate mofetil (MMF) (600 mg/m^2^ twice daily) was added alongside daily ACE-I. Tocilizumab was spaced to every six weeks after MMF initiation.

**Fig. 1:**
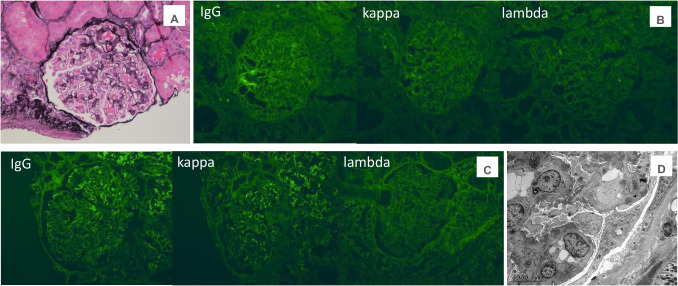
**A** Thickening of the glomerular basement membranes with abundant GBM “spikes” and “vacuoles” (Jones silver stain, × 400). **B** By routine immunofluorescence on fresh/frozen tissue, the glomeruli are negative for IgG and kappa and lambda light chains (× 400). **C** Following protease digestion of the formalin-fixed, paraffin-embedded tissue, direct immunofluorescence shows positive granular capillary wall staining for IgG and kappa light chain, and negative staining for lambda light chain (× 400). **D** Electron microscopy reveals numerous subepithelial immune-type electron-dense deposits with frequent GBM “spike” formation (× 4000)

Despite induction immunosuppression directed at C3GN, she had persistent proteinuria with UPC of 1.75 mg/mg; therefore, a second biopsy was performed. Results showed features of membranous glomerulopathy with masked IgG-kappa deposits. LM revealed thickened glomerular basement membranes with mild mesangial hypercellularity, raising suspicion for membranous nephropathy. Jones silver stain demonstrated frequent glomerular basement membrane (GBM) spikes, and several glomeruli showed segmental sclerosis. Again, no significant IFTA was noted. Routine IF again demonstrated only granular mesangial C3 staining. Given suspicion for MGMID based on her personal history of an autoimmune disease, IF was repeated on paraffin-embedded tissue with protease digestion, which revealed IgG-kappa deposits (Fig. 1).

Retrospective paraffin IF staining of the first biopsy was negative. EM from the initial biopsy showed only infrequent subepithelial deposits, likely suggesting early disease. At this time, we elected to initiate therapy with steroids and MMF for its efficacy and relative simplicity, as it does not require routine therapeutic drug monitoring. Additionally, we aimed to avoid the potential burden of an additional infusion on her quality of life.

**Fig. 2:**
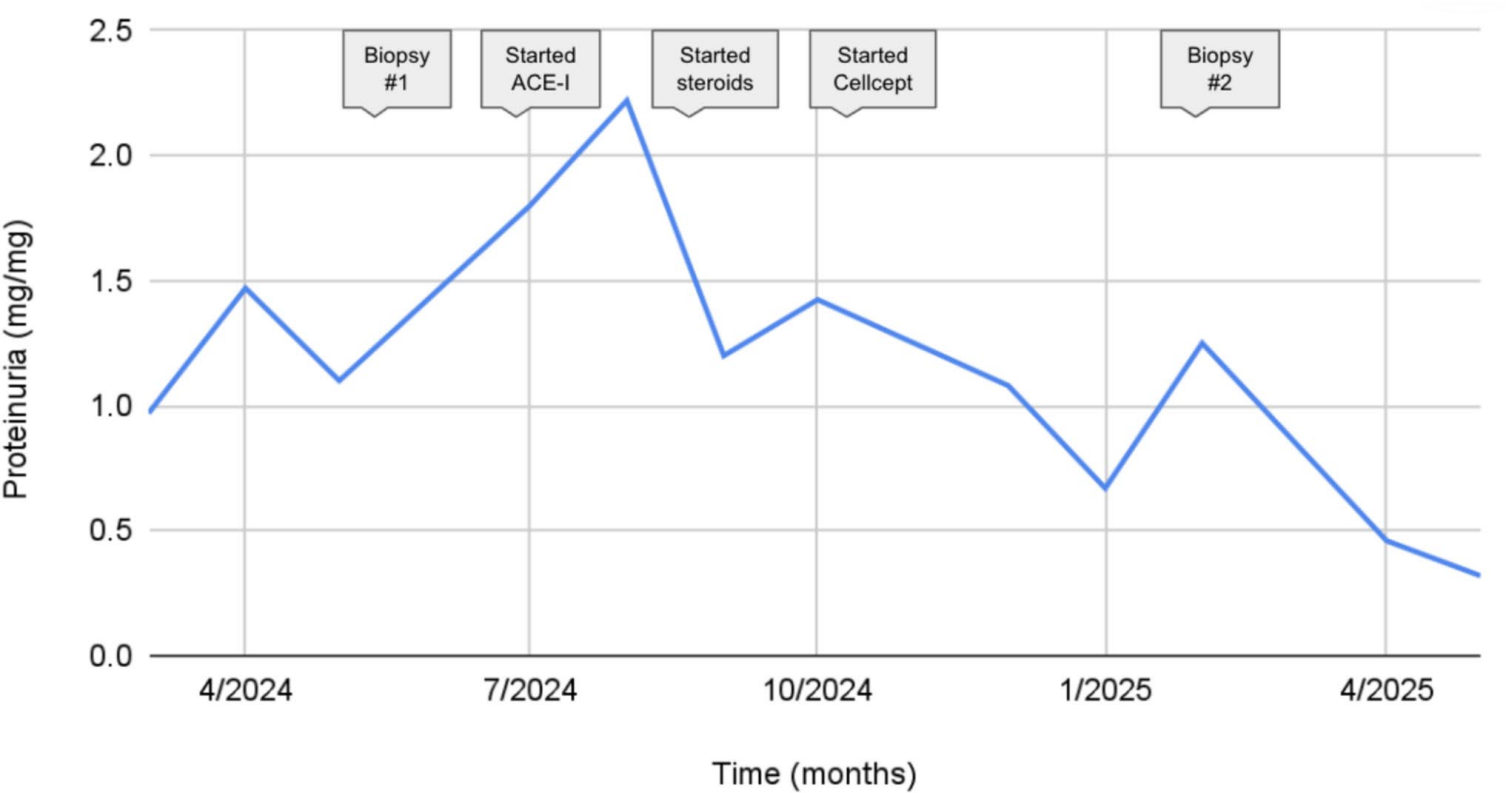
Trend in proteinuria over time in a patient with membranous-like glomerulopathy with masked IgG-kappa deposits (MGMID). Proteinuria was measured at multiple time points, beginning at initial presentation and continuing through kidney biopsy and initiation of various treatments, including an ACE inhibitor, corticosteroids, and mycophenolate mofetil

This patient has remained on ACE-I and MMF with progressive improvement, with UPC decreasing to 0.32 mg/mg after 7 months (Fig. 2). Creatinine at last follow-up was 0.47 mg/dL. Although it is unknown what her response would have been without intervention, we hypothesize that the continued resolution of her proteinuria is a result of time on treatment. Her kidney function remains normal, and tocilizumab infusions have been discontinued as her sJIA has been in long-term remission.

## Discussion

MGMID is a rare glomerulopathy predominantly affecting females with a history of autoimmune disease [1]. Diagnosis can be challenging and often delayed due to false-negative routine IF staining for immunoglobulins. The term “masked deposits” refers to immunoglobulin deposits that evade detection by standard IF, requiring repeat staining on paraffin-embedded tissue after protease digestion [4].

MGMID may mimic C3GN due to C3-dominant staining, oftentimes leading to misclassification. Therefore, when EM reveals subepithelial and mesangial deposits with minimal or absent immunoglobulin staining on routine IF and in the absence of endocapillary proliferation, paraffin IF should be strongly considered when there is high suspicion [3, 4]. Circulating antibodies implicated in membranous nephropathy were not checked; however, they are typically negative in a monoclonal gammopathy-associated membranous nephropathy.

Outcomes in MGMID are variable, ranging from spontaneous remission and mild disease to progression to chronic kidney disease (CKD) and kidney failure. Optimal treatment remains unclear, although a variety of treatment strategies have been described in the literature, including renin–angiotensin–aldosterone system (RAAS) blockade alone, corticosteroids in combination with calcineurin inhibitors, MMF, hydroxychloroquine, rituximab, or azathioprine, and in some cases, observation alone [4]. However, a combination of immunosuppression and RAAS blockade versus RAAS blockade alone appears to benefit some patients.

While MGMID has been reported to occur in association with autoimmune conditions such as Sjögren’s syndrome, very few cases of MGMID have been reported to occur in the context of an autoinflammatory disorder like sJIA. Additionally, although sJIA was clinically inactive, renal manifestations such as amyloidosis and immune-mediated glomerulonephritis have been reported, though exceedingly rare [5], therefore the role of her underlying rheumatologic disease and its control on the presence and timing of MGMID is unclear.

## Conclusions

Membranous-like glomerulopathy with masked IgG-K deposits is an emerging diagnostic entity with limited data in the pediatric population. It should be considered in adolescents and young adults with proteinuria, especially in the setting of positive autoimmune serologies or previously diagnosed autoimmune conditions and biopsy findings of subepithelial deposits with isolated C3 staining. Paraffin IF is essential to unmask these deposits and establish the diagnosis. Further studies are needed to guide treatment and define long-term outcomes.

## Summary

### What is new?

- Treatment with ACE-I and MMF was successfully used in a pediatric patient with membranous-like glomerulopathy with masked IgG-kappa deposits, resulting in a significant reduction in proteinuria and prevention of CKD progression.
